# Medical Society of the County of Erie

**Published:** 1901-06

**Authors:** Franklin C. Gram


### MEDICAL SOCIETY OF THE COUNTY OF ERIE.

Reported by FRANKLIN C. GRAM, M. D., Secretary.

THE members of this society were called to attend a special meeting in memory of Dr. Samuel G. Dorr, on April 30, 1901, in the Y. M. C. A. parlors. President William C. Phelps called those assembled to order at 12 m., and announced the object of the meeting.

On motion of Dr. A. H. Briggs, the president appointed a committee consisting of Drs. A. H. Briggs, William Warren Potter and Wm. G. Bissell, to prepare a suitable memorial. The committee presented the following, which was adopted:

### IN MEMORIAM.

Samuel Griswold Dorr, M.D., was born at Dansville, N. Y., May 30, 1840, and died at Buffalo, N.Y, April 28, 1901, after an illness of but two hours, of angina pectoris.

Suddenly stricken, while apparently in full vigor and strength of mental and physical manhood, and without the slightest warning, this death has produced a shock to the community, such as seldom before has been experienced.

Dr. Dorr, was known by thousands of persons and was respected and beloved by all. He was an active member of the Medical Society of the County of Erie, one of its most ardent supporters, seldom absent from its meetings and the society through his death has lost a most valuable and respected member.

In view of the fact that the Almighty power has seen fit to take him from our midst,

*Be it Resolved*, That this society express to the members of the family their sincere sympathy and that the members of the society attend the funeral in a body.

Signed, Albert H. Briggs,
William Warren Potter,
William G. Bissell.

Dr. A. H. Briggs delivered a fitting eulogy on Dr. Dorr, whom he had known throughout his medical career, and with whom he had been intimately associated.

Dr. W. D. Greene said Dr. Dorr was the family physician, friend and counselor, loyal to his friends and never forgot a favor.

Dr. J. J. Birmingham had practised side by side with Dr. Dorr for twenty years and testified to his great popularity in East Buffalo.

Dr. W. C. Phelps said that Dr. Dorr's inventive turn of mind stood him to good advantage in his profession, and mentioned some of the results of such work.

Dr. William Warren Potter briefly sketched Dr. Dorr's career since his graduation in medicine. He began his practice as a busy man, an unusual event for a beginner, and remained busy until his end. Later he entered the political field, holding various positions until he became postmaster of Buffalo, and as such was actively engaged at the time of his death. Withal, he was an excellent physician and one of our most respected citizens.

Remarks were also made by Drs. Callanan, Bayliss and Cott, after which the society adjourned.

